# Unintended consequences of communicating rapid COVID-19 vaccine policy changes– a qualitative study of health policy communication in Ontario, Canada

**DOI:** 10.1186/s12889-023-15861-y

**Published:** 2023-05-23

**Authors:** Elizabeth Vernon-Wilson, Moses Tetui, Agnes Nanyonjo, Maisha Adil, Arthi Bala, David Nelson, Emma Sayers, Nancy Waite, Kelly Grindrod

**Affiliations:** 1 School of Pharmacy, University of Waterloo, 200 University Avenue West, Waterloo, ON N2L3G1 Canada; 2 Department of Epidemiology and Global Health, Umeå University, Umeå, Sweden; 3 Lincoln International Institute for Rural Health, University of Lincoln, Brayford Pool, Lincoln, LN6 7TS Lincolnshire UK

**Keywords:** Vaccination policy, Vaccine hesitancy, Vaccine confidence, Vaccine inequity, COVID-19 pandemic, Public health communication, Policy change

## Abstract

**Background:**

The success of the COVID-19 vaccination roll-out depended on clear policy communication and guidance to promote and facilitate vaccine uptake. The rapidly evolving pandemic circumstances led to many vaccine policy amendments. The impact of changing policy on effective vaccine communication and its influence in terms of societal response to vaccine promotion are underexplored; this qualitative research addresses that gap within the extant literature.

**Methods:**

Policy communicators and community leaders from urban and rural Ontario participated in semi-structured interviews (*N* = 29) to explore their experiences of COVID-19 vaccine policy communication. Thematic analysis was used to produce representative themes.

**Results:**

Analysis showed rapidly changing policy was a barrier to smooth communication and COVID-19 vaccine roll-out. Continual amendments had unintended consequences, stimulating confusion, disrupting community outreach efforts and interrupting vaccine implementation. Policy changes were most disruptive to logistical planning and community engagement work, including community outreach, communicating eligibility criteria, and providing translated vaccine information to diverse communities.

**Conclusions:**

Vaccine policy changes that allow for prioritized access can have the unintended consequence of limiting communities’ access to information that supports decision making. Rapidly evolving circumstances require a balance between adjusting policy and maintaining simple, consistent public health messages that can readily be translated into action. Information access is a factor in health inequality that needs addressing alongside access to vaccines.

**Supplementary Information:**

The online version contains supplementary material available at 10.1186/s12889-023-15861-y.

## Background

Worldwide, the evolving COVID-19 pandemic has generated an array of public health and social policies along with significant scientific, ethical, and legal debate as governments have endeavored to limit the spread of the SARS-CoV-2 virus and reduce infection-related morbidity and mortality [1]. With the potential to reduce transmission and disease severity, vaccination was promoted as a pandemic exit strategy capable of returning life to ‘normal’ [2].

In Canada, pre-existing pandemic preparedness plans shaped the federal government’s response to approval, acquisition and distribution of COVID-19 vaccines, while province-specific policies defined prioritization groups and implementation plans [3, 4]. To support mass immunization, extensive guidance was developed to promote and facilitate vaccination. Health communication consisted of interpersonal and mass information sharing activities. In Ontario, public health professionals were tasked with communicating COVID-19 vaccine policy (laws, regulatory measures, guidelines, recommendations and courses of action) and delivering implementation to residents.

Designing an effective, vaccine communication strategy required audience segmentation, deployment of appropriate message content and alignment of channels to fit the communication goals [5, 6]. For example, messages needed to include clear explanations of principles guiding vaccine selection, adequate justification of prioritization criteria and a compelling narrative to create vaccine demand. At the same time, messaging needed to emphasize beneficial outcomes in culturally sensitive ways and generate a sense of human agency to combat and control the pandemic [5, 7, 8]. Despite much publicity and tailored messaging, COIVD-19 vaccine uptake has been sub-optimal in some populations, including cultural and race-based groups disproportionately hurt by the pandemic [9–11].

In Canada, as elsewhere, changing pandemic conditions have required policy makers to be agile in decision making and responsive to evolving scientific evidence and epidemiological data models. Consequently, vaccination policy and its communication transformed over time, redirecting people from protecting vulnerable individuals when COVID-19 vaccine supply was limited to emphasizing the need for community or “herd” immunity later in the vaccine roll-out [12]. However, the scientific rationale for blanket vaccination policies was challenged by the emergence of waning antibody-based immunity, new, evasive variants of concern and immunity acquired from COVID-19 infection [1]. To counter hesitancy, defined by the World Health Organization as “delay in acceptance or refusal of vaccines despite availability of vaccination services and accelerate uptake” [13], vaccination policy and messaging shifted to being more forceful. Provinces began leveraging COVID-19 vaccine requirements, mandates and passports to advance numbers required for community immunity [14, 15].

While the dynamic drivers of policy changes are understood, it is less clear what those changes felt like for those responsible for communicating policy, including local public health staff, healthcare workers and community leaders. This is particularly important as individual reactions to and interpretations of policy influence vaccine acceptance [16–18]. This qualitative study aimed to explore how COVID-19 vaccine policies were communicated, including the perception of societal response from the perspectives and experiences of frontline health and social care providers, professional advisory bodies and community organizations in Ontario, Canada. This perspective is critical to inform current and future policy around vaccine programs and pandemic responses.

## Methods

### Study context

As part of a larger study conducted in partnership with researchers at the University of Lincoln to support a comparative analysis of vaccine policy and equitable communication about COVID-19 vaccines between Canada and the UK [19, 20], we undertook a qualitative study to explore perceptions about how COVID-19 vaccine policy communication was undertaken and changed over time. Data was collected approximately one year on from the introduction of vaccines in North America and Europe. This period coincided with the start of the fourth wave of COVID-19 in Canada, driven by the Omicron variant. At this time, policy communication supported receipt of a third dose of COVID-19 vaccine and advised parents that children younger than 12 years may be vaccinated. Vaccine mandates regulated post travel quarantine requirements and access to certain public settings and facilities.

### Approach

This paper presents a qualitative description and thematic analysis of the Canadian participants' perspectives based on descriptive content analysis of interviews (detailed below). An inductive approach, together with constant comparison and reflection by research team members, shaped the generation of themes presented herein. The COREQ (Consolidated criteria for reporting qualitative research) checklist was used to guide study design, participant recruitment and data analysis [21].

### Recruitment and participant description

Participants were recruited through targeted sampling expanded by snowball sampling. Invitations to participate in a one-time online interview were sent via email to 68 potential participants representing organizations creating and communicating COVID-19 vaccine policy and guidance in Ontario. Initially, invitations were sent to potential participants identified through prior interactions with the research team at University of Waterloo. These included contacts at Waterloo Region Public Health, Ontario Ministry of Health, organizations representing healthcare professionals (HCP), health and social care provider organizations (urban and rural) and non-profit community organizations. Recipients were asked to recommend alternative contacts if they were unable or unwilling to participate in research interviews. This snowballing strategy was also used to identify and approach individuals considered to represent minority populations, be vaccine hesitant or represent the views of hesitant communities with reference to working knowledge of public health officials or through individuals’ peers’ perceptions.

From the initial pool, 29 people completed interviews from November 2021- January 2022. Interview participants included people working directly and indirectly with vaccine-hesitant groups in frontline care, service provider or advocacy roles. Informed, written consent was obtained from all individual participants prior to participation in the study, this was reviewed and reaffirmed at the beginning of each interview. Participants were offered an honorarium of $30 for their participation in the study. The University of Waterloo Research Ethics Board approved the study on 26 October 2021 (Ref: 43,633).

### Interview guide & data collection

A semi-structured interview guide was designed using the research group's expertise and informed by existing literature. It was refined following pilot interviews with participants not included in this study. Modifications led to the design of a second guide enabling questions to be focused towards elucidating the perspective of policy communicators or community recipients (guides are provided supplemental material). The guides included open-ended questions on participants' perceptions of how policy was communicated and how vaccine policy changes impacted vaccine confidence. Policy was defined in interviews as laws, regulatory measures, guidelines, recommendations and courses of action. Questions were modified or expanded to allow flexibility and help establish rapport as interviewees raised new relevant issues about their roles and experiences with COVID-19 vaccine policy communication. All interviews were conducted online by EVW, a researcher with previous experience in interviewing patients and HCPs for service evaluation. Data were recorded and transcribed using Microsoft Teams software (Version 1.5.00.6181). Interview length ranged from 40–75 min (average of 53 min). Transcripts were validated by comparison to audio recordings of interviews.

### Data analysis

An inductive and deductive approach to thematic analysis was undertaken [22]. Initially, notes on interviews were shared with other research team members to help judge when saturation was reached on subject areas. Transcripts were then validated, read, reread to improve familiarity and inductive open coding was completed on all transcripts by EVW to identify significant words, activities and responses. Constant reflection and comparison between transcripts were facilitated by discussion with team members. Open coding and organization were supported by use of NVIVO software (Version 12.7).

A subset of three transcripts selected to be representative of different viewpoints (a front-line vaccinator and clinic manager, a healthcare policy maker and a community outreach advocate) were additionally coded by three further research team members (MT, MA, BA). Comparison and collation of independently generated codes led to a single codebook. Group discussions, together with an iterative process of returning to transcripts to visualize code context, led to organization of similar codes into meaningful categories. Categories with common aspects were reviewed, considered and grouped into sub-themes and then themes such that higher order groups had substantial internal coherence and identifiable distinction between them. Codes within themes were checked for confirmation and deviation from the theme identity and cross referenced to interview memos as part of the deductive thematic analysis process. The relationship and meaning of categories, sub-themes and themes were compared until a consensus on interpretation, naming and structural organisation was reached. Researchers’ reflexivity was considered as data presentation was agreed by EVW and MT who have different but convergent backgrounds in health service systems and management.

## Results

### Participant information

**Fig. 1:**
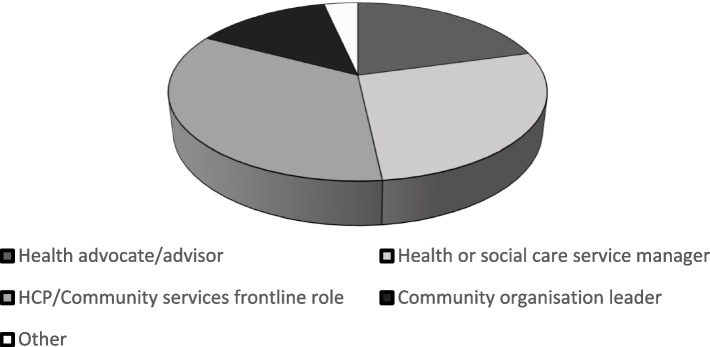
Study participants' roles in communicating COVID-19 vaccine policy

The 29 interview participants were predominantly white (76%), female (72%), with ages ranging from 23–74 years (55% were between 25–44 years). The participants roles and activities offered them varying levels of contact with the public and responsibility for communicating COVID-19 vaccine policy (Fig. 1). Some participants had regular direct interactions with clients or managerial responsibility for frontline workers making these contacts in health or social care settings (including vaccination clinics). Other participants had communication roles creating and vaccine guidance, raising awareness of vaccination opportunities and responding to queries raised. The majority of participants were in paid roles supported by professional standards for communication, though four participants had informal community roles overseeing COVID-19 vaccine policy communication.

### Findings

**Fig. 2:**
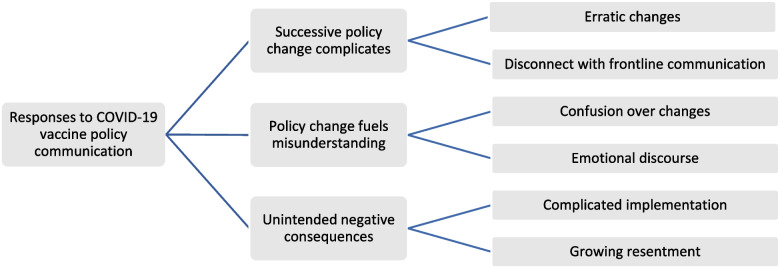
Major themes and sub-themes arising from data collected about response to COVID-19 vaccine policy communication

Participants revealed how COVID-19 vaccine policy was communicated by multi-modal dissemination including use of traditional channels such as news broadcasts and newer, social media methods. The broad use of different communication methods was seen as a strength helping communicators promote pro-vaccination messages. However, participants’ experiences of COVID-19 vaccine policy changes often elicited negative comments and expressions about the need to mitigate problems. We identified three themes in the data relating to participants’ reflections on how changes to COVID-19 vaccine policy were experienced and their perceptions of wider societal responses to the changing messages (Fig. 2). These were (1) successive policy changes complicated communication, (2) policy changes fueled confusion and misunderstanding (3) continual policy updates had unintended, negative consequences on COVID-19 vaccine confidence and uptake. Representative quotes for each theme are presented in Table 1. Throughout the dataset, participants identified both challenges and strategies that could facilitate communication about vaccine policy change.

**Table 1:** Representative participant quotes organized in sub-themes and themes

Theme/Sub theme	Representative Quotes	Participant
Successive policy changes complicate		
Erratic changes	Sometimes there were some last minute changes that made it challenging. All of a sudden we’re going to make some changes. I didn’t feel there was enough lead time for people to adjust	Non-profit social care enterprise manager
	Communicating policy, that's something we struggled with all the time. People need time to prepare. The information is so quick that two or three days after an announcement or NACI comes out with a recommendation almost feels like it's too late so we try to keep up with that in terms of relevance. You have to be constantly evaluating your policy and how you're communicating it. The environment changes very quickly around you	Professional body advocate
	When you try and do them all in rapid sequence you've got complexity of what's out of date? How quickly will we able to change online resource? We’re beginning to be out of date and the complexity was because each thing had so much volume. Pivoting to the next one was made more complex because it was almost like you had to unpick the last policy decision. That was the complexity	Director of communication
Disconnect with frontline communication	Even giving a 24 h or 48 h gap in between a change in policy and communication to the public would be very helpful, and it would really help to improve the trust that people have. Then they wouldn't be encountering those situations where policy has changed, but the actual professionals who are responsible for administering that policy don't know about the change	Pharmacy manager
	They should have said the vaccine is 95% effective for at least two months. Instead the narrative was so narrow it didn’t allow for the changing reality	Rural community outreach worker
	What the public sees is that I’m confused. The messaging changes. Also the consistency with which you are able to deliver on some of those messages is challenging	Urban pharmacist
	Information would change rapidly so it became very challenging to have updated information for everybody. Helping them communicate accurately to the community was an exercise in frustration	Non-profit social care organization director
Policy change fuels misunderstanding		
Confusion over changes	We come out with one of the first statements from any health professional group to say that we were disappointed with the messaging about changing AZ vaccine policy. Not necessarily disappointed with the science, but the way they had framed it. It was damaging and unclear and caused confusion and caused unnecessary panic	Professional body advocate (Federal)
	Initially, priority groups, as to who qualified to get the vaccine, was super confusing. It changes, it’s just never been concrete. It's just confusing	Physician
	There are of course going to be those who are really confused and concerned because the news may be different to what we're saying, but it's simply because those students, those patients are not up to date with the most [recent] NACI guidelines or whatnot or Health Canada's recommendation	Nurse practitioner
Emotional discourse	The time between vaccines changed, people were like ‘Why?’ Why are you coming out telling us very strongly this is the vaccine sequence and now you changed it. What am I supposed to believe? Do you actually know what you're doing?	Non-profit social care enterprise manager
	Trying to tell people that they had to get one Astra Zeneca and then an mRNA or the mixed mRNA [vaccines]. That was that was brutal. It was so frustrating because people were so against it. I don't know what they were believing. They were just thinking that we were going to try and kill them	Nurse practitioner
	The mixed dose regimens and keeping people from travel; change is another thing that has caused a lot of people angst	Vaccine clinic manager
Unintended negative consequences		
Complicated implementation	It would be nice to have had a heads up on things prior to press releases. Oh my God, that would have helped us a lot	Community pharmacist
	Eligibility criteria were slightly different from province to province. The vaccines were rolled out in pharmacies at different rates in different provinces	Professional body advocate (Federal)
	The way the province regulates intervals for the indigenous community was they had urban indigenous and rural Indigenous. They changed the dose interval at one point just for urban Indigenous. Three weeks later, the provincial guidance changed so we had to rework all of our clinics	Vaccine clinic team lead
	I would tell you that the vaccine rollout has been challenging because the rules keep changing. It's been that way from the start. Most of that has come down from our provincial government around who is eligible and who can get the vaccine [and] when have been very hard to enact. We never get enough notice so that we can't plan. There's no planning in this and it's incredibly confusing for the general population	Vaccine clinic manager
	The poor people, they're probably scrambling to get all this together so that by Monday morning we've got what we need to tell us the new parameters. Another thing was when you get your booster shots, so the change in duration between shots. That policy keeps like flipping out and changing	Rural community pharmacist
	When guidance gets announced there’s a catch up. We don't have enough time to plan and to make sure that we are really ready to go when these policy announcements happen…It became chaotic	Professional body advocate (Provincial)
	Vaccine passports, how those policies [worked], that was one of the most nerve-wracking times for our team about clarity. That’s a whole thing we continue to navigate	Non-profit social care organisation director
Growing resentment	A lot of people have questions about the vaccine where they feel like they’ve been lied to. It’s because the reality shifted and the communication didn’t shift in lockstep with it. It took a while for communication to catch up and during that period a lot of cracks emerged and people lost trust with a lot of policy in general	Rural community outreach worker
	I think when everything is so up in the air, the one thing that we really need is consistent messaging and if NACI, is saying one thing, and healthcare is saying another and then Ontario does its own thing, I think that's going to cause people a lot of confusion and a distrust towards the system as a whole. Waiting for information to filter down is extraordinarily infuriating	Nurse practitioner
	Policy communication? Confusion frustration yeah, eventual lack of trust, inconsistency, constantly changing these are more terms, but these are things that I feel like we were hearing from people coming to us, yeah	Public health worker

#### Successive policy changes complicated communication

Interview participants with different roles in communication described how difficult it was to keep step with rapid COVID-19 vaccine policy changes. Two subthemes were derived from categorized codes; *erratic changes* and *disconnect with frontline communication*.

Vaccination policy was widely communicated to promote public awareness, but the timing of updates sometimes led them to be viewed as *erratic changes.* The number and frequency of updates led to simple messages becoming more complex as eligibility criteria and COVID-19 vaccine information became more nuanced over time.

*“The highly changeable circumstances led to simple policies being more difficult to communicate because they changed so quickly. When you do them in a rapid sequence, you’ve got complexity--what's out of date?” *(Director of Communication)

The need to provide prompt updates with adequate explanation of policy changes, alongside business-as-usual activities, stretched organizations’ resources. Deploying innovative approaches such as shared video messages was reported to be helpful in reaching staff, volunteers and clients, however this demanded skill and time. Needing to manage policy versions and frequently update a wide range of information materials were named as exhausting stressors born out of policy changes. This was expressed by a vaccination team leader, who provided materials designed to meet the local populations’ literacy, linguistic and cultural needs and a lead hospital physician,

*“We got out what we could, where we could, as fast as we could, then everything else lagged behind. I wish we had more resources to make it consistent across the board…it felt difficult to say to people, ‘Oh, the translations are coming’.” *(Vaccination Clinic Lead)

*“The hardest thing has been just how quickly everything changes and how it's often hard, too hard for people to keep on top of the latest iteration.” *(Rural Hospital Physician)

Gradually, the imperative to update swiftly was softened by repeated policy changes. However, because changes were erratic, it was hard to plan a meaningful schedule for reviewing vaccine information for accuracy. Consequently, participants described how gaps in the communication chain frequently emerged. Inequalities in information access also became apparent. Participants felt communication gaps were often filled with data from international sources, some of which did not align with Canadian policy.

*“A lot of the information they get is from their news channels, directly from India, Pakistan or wherever they're from. A lot of the information from there doesn't actually coincide with what's happening here.” *(Urban Hospital Physician)

*“Not all areas, or public health units, or municipalities have the capacity to translate information. You end up with piecemeal information that's translated and being shared without people knowing the source of the information or the accuracy of the information.” *(Public Health Community Outreach Worker)

A second subtheme, *disconnect with frontline communication,* arose from comments about how frontline workers felt responsible for explaining COVID-19 vaccine policy changes to the public. Those with frontline roles described how the reasons for policy changes could appear opaque or heavily reliant on literacy skills, internet access and familiarity with English. One pharmacy manager described the added burden of trying to paraphrase vaccination recommendations in simple terms and provide a rationale each time a policy was refined or changed.

*“The most difficult thing to communicate was helping patients navigate the different criteria and eligibility and helping health professionals understand that. It was a very complex the way we went about rolling out the vaccines. It made it complicated for people to understand and made it very complicated for pharmacies and pharmacists to understand and keep track of and to stay up to date.” *(Community Pharmacist)

*“The biggest challenge is that the rules change every day, all the time. How do we communicate that clearly to everybody and help make people understand? Obviously, we try our best but if somebody doesn’t watch the news, read the paper, listen to the radio, how do we get that message to them?” *(Regional Vaccine Clinic Manager)

Online web materials and webinars were felt to be helpful education resources, but representatives of healthcare professional bodies were among those commenting on the struggle to quickly prepare supporting information and synchronize its release to match new policy positions. Lack of synchrony was felt to hinder the adoption of best-practice ideas.

*“There are times when we find out a few hours before. There’s not enough time to make sure we are ready to go when these announcements happen…In terms of eligibility, that has changed more times than I can count...it feels like roughly weekly, we’ve issued a new communication.”* (Provincial Professional Body Advocate).

#### Policy changes fuelled confusion and misunderstanding

In the wake of multiple, rapid policy changes, confusion about COVID-19 vaccine recommendations grew in ways participants felt were frustrating and avoidable. This led to the sub-themes of *confusion over changes* and* emotional discourse.*

*Confusion over changes* arose in part because four areas of COVID-19 vaccine policy change proved challenging to navigate with certainty: priority groups, dosing intervals, recommended COVID-19 vaccine brand and COVID-19 vaccine requirements/ mandates.

Another issue was presenting guidance that appropriately dealt with regional COVID-19 vaccine supply, local infection rates and was consistent with national recommendations. The need for cohesion and co-ordination is described here by a director of an organisation representing HCP across Canada and a vaccine clinic manager.

*“We pointed out to the federal government that each province is coming out with a completely different level of eligibility; age ranges were different from province to province. We tried to help bring cohesion.”* (Federal Professional Body Advocate)

*“Whenever the province is not giving us clear policies and clear mandates and local regions must make decisions, it is very, very, challenging for us to deal with it. Then of course it is more confusing for people, right?” *(Regional Vaccine Clinic Manager)

Provincial variations and political decision-makers’ influence on messages led to the trustworthiness of information sources being questioned. Participants spoke of confusion mounting and the need for consistency and transparency.

*“Political decision-making hasn't really been in tune with what the health care professionals are saying... confusingly, the doctors were saying this, but [the Premier of Ontario] is doing that. So, what's really going on?.........for vaccines or mandates, it should all be evidence-based because your evidence ‘for’ is pretty consistent…. We wouldn't have as much trouble. Our policies are swindled by political decisions or pressures from business.” *(Urban Hospital Physician)

The politicizing of COVID-19 vaccine policy was one component in the sub-theme *emotional discourse.* Amid the pre-existing stress of the pandemic, the volatile policy environment contributed to information turbulence and unsettled interactions between the public and HCPs. For example, this clinic manager spoke of emotional responses to mixed vaccine policies,

“*There was so much angst and anger and upset about that because people so desperately wanted to get the vaccine and didn't have trust that getting a second dose of Moderna was going to be safe and effective and allow them to travel.” *(Regional Vaccine Clinic Manager)

Policy change became an additional stressor to frontline workers who already felt the need to continually fact-check details and confirm their understanding was up to date when communicating with the public. This pressure contributed to anxiety and stress within healthcare teams.

*“I've struggled with just being in my profession now because I've always prided myself on being a resource for people. It took me many, many months to not feel anxious because of all those changes. I just felt, ‘What if I say the wrong thing?’ or ‘Am I going to catch up?’ You can barely catch a thought before the phone rings again.”* (Rural Community Pharmacist)

HCPs described a hidden communication burden associated with policy change as they struggled to match what they could offer within current vaccine policy guidelines to people’s expectations. There was a sense that this damaged relationships between HCP and clients.

*“It added so much stress because patients were calling, and pharmacy teams didn't have answers. It fractured the relationship and the trust, especially in an environment that was already so confusing and stressful and layered with this information and personal beliefs.” *(Pharmacy Association Advocate)

Many frontline staff also spoke of positive emotional discourse; team camaraderie in dealing with stressful times, gratitude and appreciation from people receiving vaccinations.

“*Our patient population has been extremely appreciative of the messaging and timely response, of doing what we’ve been able to do to get them vaccinated as soon as possible.” *(Nurse Practitioner)

#### Policy changes had unintended negative consequences

The final theme links two sub-themes, that policy amendments led to *complicated implementation* and *growing resentment*.

For healthcare providers, the practical need to keep up to speed with successive policy changes *complicated implementation.* It meant learning to rapidly adapt implementation plans and messaging. Uncoordinated communication at any point would potentially muddle the already complicated logistics of COVID-19 vaccine distribution and administration.

“*Every time they open up a new age group of eligibility, suddenly we have to figure out, OK, now we're going to have to open up more vaccine clinic time slots. Who's going to staff that?” *(Family Physician & Hospitalist)

Announcements or press releases were often made during daytime working hours, which meant busy HCPs were often brought up-to-speed by members of the public who had heard the most recent change prior to arriving for a vaccine appointment, or who were calling for the clinic or pharmacy for more information. Significantly, HCPs like the pharmacist and physician below, felt patients’ confidence in their professional judgement and ability to deliver other healthcare services beyond vaccines were eroded by these circumstances.

*“It can look a lot like nobody knows what they're talking about. Last week they said it was like this, and now they're saying it's like that. It’s unsettling for people and shook their confidence.” *(Family Physician & Hospitalist).

This intensified workplace stress and fatigue stemming from operational challenges and led to *growing resentment* and frustration with the institutions creating policy. This pharmacist recalls feeling foolish and inadequate as a service provider as a consequence of complicated policy communicated poorly.

*“At times, we look like we don't know what we're doing, whether that's between the policies being too complicated or not communicated with enough time, or not flowing the way they're intended to flow. We look foolish, and we’re disappointing people. That has a negative impact on how they perceive us….It’s frustrating. Then, you get frustration from the patients. Yes, I got yelled at” *(Community Pharmacist)

Community outreach funded by emergency funds helped reach vulnerable ethnic minorities. However, the roll-out of COVID-19 vaccine was complicated by the constantly changing narrative. For example, Indigenous communities were among the first to be offered COVID-19 vaccinations in Canada, but message instability about vaccine schedules roused confusion, disquiet, and mistrust. Policy amendments thus frustrated groups trying to engage with minority populations.

*“We did have…some anger or pushback when the dose intervals changed. The way the province regulates dose intervals for the Indigenous community - they had Urban Indigenous and Rural Indigenous. They changed the dose interval at one point just for Urban Indigenous… three weeks later, the provincial guidance changed, so we had to rework all of our clinics.” *(Vaccine Clinic Lead).

*“The changing messaging and community not understanding why decisions were made in the first place…that caused a lot of confusion and potentially mistrust.” *(Public Health Community Outreach Worker).

Resentment and frustration with COVID-19 vaccine policies were felt to build where choice and self-determination were jeopardized. This perception was expressed by a participant interacting with a strict religious community,

*“It’s still a choice in Canada. If it as ever law they would get vaccinated but when it's their choice, they would feel that it's coming from government and they would be quite hesitant.” *(Community Care Manager)

Low- income public service and healthcare workers were among those seen to be pressurized to get vaccinated. Interview participants who had direct contact roles as vaccinators or through line-management reported some workers were resentful. However, levels of sympathy varied as COVID-19 vaccination requirement policies in healthcare was seen as too flimsy by some participants and as an assault on civil liberties by others.

*“Hospitals are restricting unvaccinated visitors from coming in, but our government isn't giving any directive in senior-living. We’re required to still let unvaccinated visitors come in.” *(Long-term Care Home Manager)

*“There are ones who are saying ‘I’m not convinced, if I play into it then I’m just adding to the problem of government control” *(Non-profit Health Communications Expert)

*“In unionized environments, the unions were like ‘No! You can’t do that as an employer!’” *(Urban Physician)

The flexibility of vaccine guidance became another source of frustration and disunity. While policymakers aimed to provide an adaptable framework, the on-the-ground reality was that the variable rules led to discontent and confusion. COVID-19 vaccination requirements were believed to be helping increase immunization rates, but rules such as mandates or passports often conflicted with the open-door policies of many non-profit organizations and religious communities and interfered with their abilities to support their communities. Disillusionment with COVID-19 vaccine guidance was further attributed by participants with community facing roles to the perceived fallacy that vaccination would relieve all public health restrictions and return life to normal.

“*A year ago, we were all gung-ho because we were going to be able to be vaccinated and then everything would be fine again. That hasn't been the outcome at all.” *(Community Group Coordinator)

Failure to inform the public about what COVID-19 vaccines could realistically deliver was considered to have led to community held misconceptions about a post-vaccination world and eroded confidence in public health advisories. Some participants felt this might have a future negative impact on vaccine confidence generally, as expressed by this community outreach volunteer and this physician.

*“A lot of people became vaccine negative because they felt like the vaccine no longer provided them with what they thought it was going to. They felt tricked or something like that. I even got this from vaccinated people when I was talking to them.” *(Community Outreach Worker)

*“They are now going to be more mistrustful of other types of vaccines. Just because of how much a part of their political and social identity has become marked by their stance on COVID vaccination or their objection to being coerced into getting this vaccine.” *(Family Physician & Hospitalist)

## Discussion

The COVID-19 pandemic saw federal, provincial, and institutional policymakers face considerable challenges in making and communicating effective health policy to protect the population from the most severe consequences of COVID-19 infection. Decisions were often made quickly in response to limited information and updated as novel data became available. To the best of our knowledge, this research was the first qualitative exploration of how COVID-19 vaccination policy was communicated and responded to from various stakeholders’ viewpoints in the Canadian context.

Health communication is a well-established discipline, and extensive research on health promotion points to the advantage of targeted and tailored communication [2, 5]. The necessity for deliberate, explicit, and consistent policy and communication in challenging times has also been identified [6, 23]. However, the findings of this study demonstrate another consideration that is not well described in the literature—that there is a critical need for policy makers to strike a balance between continually fine-tuning policy under changing circumstances and creating policy that allows simple, consistent messages during a crisis.

Kumar and colleagues [24] describe ignorance and confusion as inevitable phases of vaccine hesitancy. In our study, interview participants agreed that policy amendments were necessary but also felt that the evolving communication complexity led to confusion in their circles and limited their ability to provide clear and accurate communication to the public. Confusion, a common expression in our dataset, was linked by those with frontline vaccine communication activities to emotional discourse, heightened feelings of uncertainty, insecurity and weakened confidence in vaccines and systems that promoted them. This risk, that confusion will fuel vaccine hesitancy, means that policymakers must scrutinize COVID-19 vaccine policy updates and communication strategies to optimize message consistency and explain with candor and empathy the actions being taken to reduce uncertainty [25]. Clarity around the rationale for policy change was felt to be needed by HCP too, a finding that resonates with other studies that have similarly demonstrated policy influences clinical practice, HCP wellbeing and patient trust [26, 27]. Their perception was public trust in them as HCP, and COVID-19 vaccines was eroded by constantly tweaked recommendations and the lack of clarity about when new policy directives could be implemented.

While laudably delivering COVID-19 vaccine information in different languages, health providers struggled with timing and volume. In our study, a key side-effect of rapid changes to policy content was that it eventually stretched the resources for translations, leading to critical time gaps in producing culturally relevant COVID-19 vaccine information materials. Access to health policy information is an ethical obligation that affects individuals' right to autonomy and capacity to make informed decisions about their welfare [28]. Study participants acknowledged bold efforts were made to address culturally and linguistically diverse communication needs, but the gaps left by delayed translations were often filled with unreliable lay advice, rumors, or international information that differed from Canadian guidance. A tsunami of COVID-19-related false information has been produced through the pandemic [29]. By creating communication gaps, rapid policy updates potentially exacerbated COVID-19 health disparities of race, class, language, and place and undermined vaccine confidence [30–32]. Early steps taken to engage with Indigenous communities and prioritize their vaccination have helped achieve good vaccination rates [4]. However, community engagement efforts were often frustrated by changes to key messages as policy changed around preferred vaccine type and dose interval. In our study, some ethnic minority groups, already concerned by misinformation about vaccine safety and the rationale behind selection of priority groups, felt angry about being misled and fearful that this was evidence of systemic racism. Their mistrust speaks to the need for full disclosure and transparent explanation when vaccine recommendations are modified. Our findings may also additional context for why some racialized and disadvantaged communities had more vaccine hesitancy than others [9, 11, 33].

This final point is representative of the unintended consequences of continued policy fine-tuning – how frequent vaccine policy changes disrupt vaccine roll-out. Frontline healthcare providers gave stark commentaries about the emotional burden of delivering vaccination programs in busy environments where increasingly complicated and constantly changing policy became an additional stressor rather than a lever to enact equitable vaccine provision. How HCP experience policy changes is an essential point because of their pivotal role in influencing patients’ vaccination choices [27, 34, 35]. Individuals involved in the on-the-ground vaccine rollout need time to understand to changes, translate and adapt operations, and adjust communication—all before facing patients.

Many communication problems with rapidly changing COVID-19 vaccine policy stem from the complexity of policy making systems and their remote distances from target audiences. Time elapses as communication trickles to frontline responders and many communication channels are contingent on opt-in alerts, membership in professional bodies or other organizational gatekeepers. To ameliorate the effects of fragmented, layered systems, communication strategies must be developed, tested and routinely used in non-pandemic conditions if we expect them to work in pandemic conditions. Rapid communication routes to frontline providers of vaccination and care are essential if delays and misunderstanding are to be avoided. The findings of this study also suggest that restraint, and checks and balances should be adopted before changing vaccine policy again and again to limit negative consequences. High thresholds may need to be met before policy change is warranted in quickly evolving situations such as this pandemic. There is a risk that modifying vaccine recommendations for one population leaves another feeling confused, frustrated or underserved. As protocols addressing use of the various COVID-19 vaccines were approved, recipients of early vaccines and protocols had cause to question whether they got the best, safe and efficacious deal. Transparent information sharing and open recognition that the best course of action may change over time could go some way to supporting trust in vaccine policy decisions.

A third recommendation from our findings is that diversity amongst policy makers and direct contact with highest risk communities and service providers are essential if adaptations to policy are intended to fit and serve populations well.

This qualitative study is the first to assess how Canada's COVID-19 vaccine policy changes were communicated and responded to. Our study is constrained by its sample being confined to Ontario, its restricted reach into highly vaccine hesitant groups and limited direct contact with those experiencing significant barriers to vaccination such as inadequate supply. Whilst efforts were made to approach individuals with diverse views and backgrounds, not every potential participant responded and their reasons for declining to participate were not successfully collated. A longer study period may have improved opportunities for additional follow-up in this area. Participants' recall of responses to policy change may be affected by frequency, impact or alignment with their beliefs. Recalled responses may represent conflation of personal experiences with the narratives of others. These limitations could contribute to bias in our data. In addition, the design of public healthcare models, accessibility to healthcare services and COVID-19 vaccine provision are important determinants of policy, communication and societal response elsewhere in the world. That said, we believe that this study has led to the generation of a rich qualitative dataset that provides unique insight into the experiences of policy communicators and community leaders during a global crisis.

## Conclusion

This study illustrates how frequent, rapid vaccine policy changes complicate vaccination communication and administration. Coordinated communication strategies are needed to support public health messaging and would benefit from frontline user feedback. Evolving pandemic conditions make policy updates necessary, but policymakers must be mindful of unintended consequences when they seek to change and improve policy. Emphasis must be placed on transparency and the preservation of core messages to avoid ambiguities and subsequent damaging responses like confusion and frustration, which may lead to vaccine hesitancy.

The results also indicate that a careful consideration of resources needed to support policy communication, such as strategies for updating official translations for each change, are required. Community engagement takes time and resources, and policy makers must be very careful that any policy changes do not ongoing efforts to build vaccine confidence in diverse communities.

## Supplementary Information

**Additional file 1.** Interview Guides.

## Data Availability

Reasonable requests for access to original data can be made to E Vernon-Wilson.
