# Supplementary material for: Unintended consequences of communicating rapid COVID-19 vaccine policy changes– a qualitative study of health policy communication in Ontario, Canada
Source: BMC Public Health. 2023 May 23;23:932. doi: 10.1186/s12889-023-15861-y (PMC10203676; doi:10.1186/s12889-023-15861-y)
Supplement: Supplementary file 1 — Additional file 1. Interview Guides. [file 12889_2023_15861_MOESM1_ESM.docx]

**Supplementary Information- Interview Guides**

**Item 1- Interview guide for policy communicators**

*To understand community level efforts that are being made to build vaccine confidence among rural and urban hesitant groups of people.*

*To understand the different communication channels, methods and messages used to convey COVID-19 vaccine policy, changes over time, and community and societal responses to these communications.*

1. What are your views about vaccines in general?
   - What has influenced your views on vaccines?
   - What are your views (confidence etc.) about COVID-19 (and other) vaccines more generally?
   - Are there any vaccines that you trust more than others? If yes, please explain
2. What are some of the efforts being undertaken in your community to increase vaccine confidence among more hesitant people?
   - Which of these efforts have been most successful and why?
   - Which have worked less well and why?
3. Could you please tell me what your role has been in policy level communication related to COVID-19 vaccination? ***NB (by policy I am referring to laws, regulatory measures, guidelines, recommendations and courses of action)***
   - What are some of the policies you have had to communicate about COVID-19?
   - When and to whom were you required to communicate these policies?
   - Where there any policies that were more difficult to communicate? Which ones in particular and why?
   - Did you have to adjust or change the type of message you were communicating? If yes, why were you required to make these adjustments?
   - Could you give examples of when you were required to change the type of messages you were providing
   - What did you think and feel about these changes?
4. What are some of the channels or methods that you have used to communicate the different policies to different stakeholders or groups of people?
   - How did you decide on the type of method you used to communicate COVID-19 vaccination policy?
   - Which channels, if any, were more effective than others and why?
   - Did you use different methods of communication for different groups of people and why?
5. What response or reaction did you receive from the public in relation to your policy communication?
   - How did you deal with more negative responses/reactions?
6. Have you at any time received questions about COVID-19 vaccine policies?

- What are the questions you received? When and how often did you receive these?

**COMMUNITY IMPACT**

1. In your experience, how do you believe COVID-19 vaccination policies should be best communicated to your communities and society?

- Whose responsibility would this be?
- Why do you believe this is the case?

1. What impact do you think communication about COVID-19 vaccines will have on the uptake of other vaccines in your community?
   - Positive and negative outcomes of the communication?
2. If you were to change your communication strategy, what are some of the things you would change?
   - Why would you make those changes?
3. If you were to improve or change your efforts to build vaccine confidence among more hesitant individuals or groups, what changes would you make?
   - Why would you make those changes?

**Item 2- Interview guide for community members/leaders**

Research objective: To understand community level efforts that are being made to build vaccine confidence among rural and urban hesitant groups of people.

**COMMUNICATION ABOUT VACCINES**

1. What are your views about vaccines in general?
   - What has influenced your views on vaccines?
   - What are your views (confidence etc.) about COVID-19 (and other) vaccines more generally?
   - Are there any vaccines that you trust more than others? If yes, please explain
2. What COVID-19 vaccine related messages, laws, or advice have you heard about?

- What are the messages that you heard most strongly?
- Where and when did you hear these messages, laws or advice?
- Who communicated the messages, laws, guidelines or advice?
- What did they mean to you and how did you feel about these laws, messages or advise?

1. Did any messages or communication you received about COVID-19 vaccines and vaccination change over time?

- If so, how did it change?
- What did the changes mean to you and how did you feel about these changes?

**COMMUNITY IMPACT**

1. What impact do you think communication about COVID-19 vaccines will have on the uptake of other vaccines in your community?
   - Positive and negative outcomes of the communication?
2. What are some of the efforts being undertaken in your community to increase vaccine confidence among more hesitant people?
   - How do you feel about these efforts?
   - Which of these efforts have been most successful in your opinion and why?
   - Which ones do you feel have not worked so well and why?
3. If you were to improve or change your efforts to build vaccine confidence among more hesitant individuals or groups of people, what changes would you make?
   - Why would make those changes?
